# Bioprinted vascular tissue: Assessing functions from cellular, tissue to organ levels

**DOI:** 10.1016/j.mtbio.2023.100846

**Published:** 2023-10-28

**Authors:** Haihong Jiang, Xueyi Li, Tianhong Chen, Yang Liu, Qian Wang, Zhimin Wang, Jia Jia

**Affiliations:** a School of Life Sciences, Shanghai University, Shanghai, China; b Sino-Swiss Institute of Advanced Technology, School of Micro-electronics, Shanghai University, Shanghai, China; c Shanghai-MOST Key Laboratory of Health and Disease Genomics, Chinese National Human Genome Center at Shanghai (CHGC) and Shanghai Institute for Biomedical and Pharmaceutical Technologies (SIBPT), Shanghai, China

**Keywords:** Bioprinting, Bioink, Blood vessel, Vascularized structure, Assessment of function

## Abstract

3D bioprinting technology is widely used to fabricate various tissue structures. However, the absence of vessels hampers the ability of bioprinted tissues to receive oxygen and nutrients as well as to remove wastes, leading to a significant reduction in their survival rate. Despite the advancements in bioinks and bioprinting technologies, bioprinted vascular structures continue to be unsuitable for transplantation compared to natural blood vessels. In addition, a complete assessment index system for evaluating the structure and function of bioprinted vessels in vitro has not yet been established. Therefore, in this review, we firstly highlight the significance of selecting suitable bioinks and bioprinting techniques as they two synergize with each other. Subsequently, focusing on both vascular-associated cells and vascular tissues, we provide a relatively thorough assessment of the functions of bioprinted vascular tissue based on the physiological functions that natural blood vessels possess. We end with a review of the applications of vascular models, such as vessel-on-a-chip, in simulating pathological processes and conducting drug screening at the organ level. We believe that the development of fully functional blood vessels will soon make great contributions to tissue engineering and regenerative medicine.

## Graphical abstract

**Figure:**
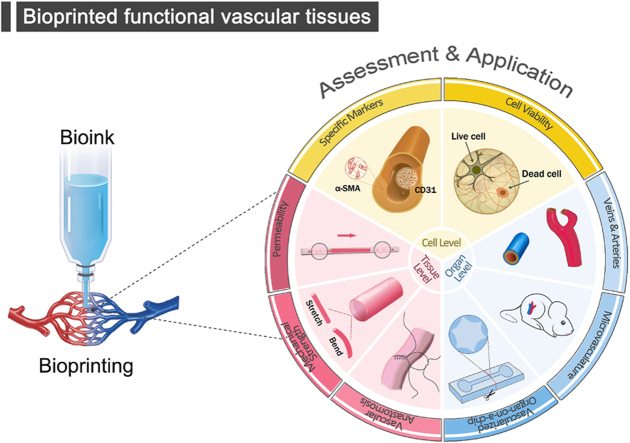

## 1 Introduction

Recently, bioprinting technology has become an essential role in the field of tissue engineering. This technology allows for accurate cell placement in specific locations to develop clinically relevant bionic constructs [1]. Particularly in the area of vascular fabrication, encompassing functional vessels and networks plays a vital role in the survival and successful transplantation of engineered organ tissue structures. In vivo, blood vessels form various patterns to optimize nutrient transport and oxygen exchange. Rapid and long-lasting vascularization is crucial to sustain viability and functionality of engineered tissues [2].

In vascular engineering, fabricating large-diameter vessels is relatively straightforward; while challenges abound when it comes to crafting small-diameter vessels, such as small arteries, veins, and capillaries. The complex capillaries pose a particular challenge for in vitro reproduction, making fabrication methods highly demanding. In tissue engineering, there are two primary strategies to create vessels through bioprinting: the bottom-up and top-down approaches [3,4]. Table 1 provides a comprehensive comparison of these two bioprinting strategies, outlining their respective fabrication targets, advantages, and disadvantages. The top-down method involves the pre-design of structures; while the bottom-up approach relies on cellular and extracellular cues to foster the development of native vascular networks [5,6]. Traditional vascularization typically employs a top-down approach wherein cells are seeded onto bioprinted scaffolds [7]. However, hierarchical vascular networks formed through bottom-up approaches face limitations in large scale tissues due to cell death resulting from hypoxia and the protracted time required for the spontaneous formation of vascular networks [8]. Combining elements of both bottom-up and top-down approaches may hold the key to replicating complex vascular systems at various scales.

**Table 1:** Comparison of top-down and bottom-up approaches [5,7,8].

Bioprinting approach	Definition	Factors affecting vascularization	Advantage	Disadvantage
Bottom-up	Regeneration: cellular and extracellular stimuli to promote vessel formation	GFs/sustained-release/oxygen gradients/morphogenesis/blood flow	Spontaneous formation of a natural vascular network	Prolonged formation of vascular network/formation of dense and twisted structures
Top-down	Reconstruction: pre-design the vasculature	Biophysical and biochemical cues/geometric elements	Reproducing/simulating vascular structures at multiple scales	Uneven distribution of cells/uncontrollable orientation of alignment

Both these two vascular fabrication approaches are essentially angiogenic remodeling and biofabrication strategies. They require the synergistic actions of bioink formulations (providing structural and mechanical support and maintaining cell viability) and bioprinting technologies (offering a platform for fabricating transplantable vessels and vascularized structures). For the fabrications of the vascular network composed by capillaries (critical for oxygen and nutrient delivery [9]), bioprinting technology has great potential to precisely build tissues with endogenously generated vascular networks, or to facilitate the invasion of vessels into printed constructs to generate capillary networks. For large/small diameter vessels, the focus of reconstructing the vascular system is to directly print vessels to mimic the structures and functions based on their target functions in vivo, rather than simply reproducing their hierarchical structures [6].

**Fig. 1:**
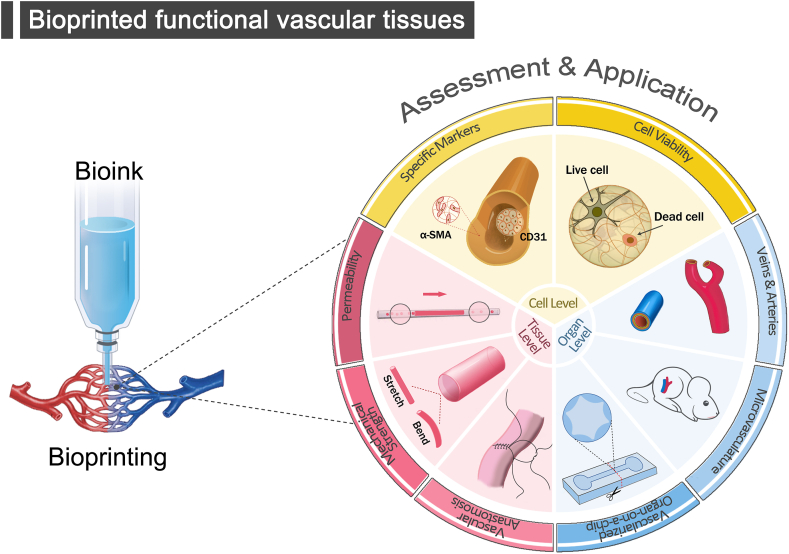
**Function of bioprinted vascular tissues.** Assessment and application at cellular, tissue and organ levels.

However, bioprinting has not yet been able to reproduce constructs that have the exact same compositions and structures of the natural blood vessels, not to speak of the comprehensive biological functions of them. Importantly, there are limited investigations about the systematic assessments of the structures and functions of bioprinted vessels or vascular systems from the view of vascular physiology. Therefore, this review starts with the selections and the importance of bioinks and bioprinting technologies for fabricating vascular tissues. Next, vascular functions are assessed in terms of both vascular constituent cells and vascular tissues, respectively. High cell viability is a prerequisite to initiate vascular function, and the expressed protein markers are capable of indicating the vascular tissues types, the development stages and the developmental morphologies. In addition, vascular functions can be assessed in vitro/in vivo based on the physiological properties possessed by the blood vessels. The functions of blood vessels such as permeability and mechanical properties are noteworthy in vitro, and the success of vascular grafts also depends on the efficient and rapid integration of host cells within the graft in vivo [10]. Finally, the application of vascular structures to different biological scenarios based on different functions is outlined in this section, which focuses on biomodels of vessels combined with various types of organs, as well as models (such as disease models) in combination with organ-on-a-chip, with potential applications in other vascular simulation scenarios in the fields of tissue engineering, drug screening, and regenerative medicine (Fig. 1).

Neither physiological functions nor morphological structures of bioprinted blood vessels is comparable to those of natural vessels, so their detectable physiological indicators may differ from actual markers from physiology vessels [11]. Nonetheless, assessment at these three levels provides valuable insights into the biological function and potential applications of artificial blood vessels for in vitro disease studies and grafts for in vivo therapies.

## 2 Bioink

The successful bioprinting of biomimetic vessels hinges on the development of bioink characterized by specific rheological properties, biocompatibility, and vascular mechanical properties [11]. Bioinks encompass a blend of biomaterials and living cells, incorporating bioactive factors such as growth factors (GFs), cytokines, and exosomes [12], which serve a pivotal role in emulating the natural tissue microenvironment, fostering cell growth, proliferation, and self-assembly of microstructures [9]. These functions are imperative prerequisites for the bioprinting process, contributing significantly to reconstruct vascular structures [13]. With updated bioink formulations, vascular tissues of different sizes have been fabricated based on the mixing of multiple types of cells from a variety of biomaterials for bioprinting (Table 2).

**Table 2:** Bioinks for constructing different vascular structures.

Types of vessels	Bioink		Ref.
	Biomaterials	Cells	
Large caliber vessels (mm-scale)	Alginate/montmorillonite/nanocomposite	NIH-3T3 cells	[14]
	Gelatin methacryloyl (GelMA)/methacryloyl/recombinant human tropoelastin	Cardiomyocytes (CMs)/Cardiac Fibroblasts (CFs)/HUVECs	[15]
	GelMA	HUVECs/SMCs	[16,17]
	Fibrinogen/collagen/gelatin		[18]
	Nanosilicate/GelMA/PEGD/ECM		[19]
	Fibrin/polyethylene glycol (PEG)/polytyramine/gelatin	Human skin fibroblasts (HDFs)/HUVECs	[20]
	Acetylated gelatin/hydroxyapatite	Dermal microvascular Endothelial Cells (HDMECs)/Human Adipose-derived Stem Cells (ASCs)	[21]
	GelMA/Hyaluronic acid (HA)/glycerin/gelatin	HUVECs/SMCs	[22]
	Collagen/Methacrylate	Human Adipose Microvascular Endothelial Cells Dental Pulp Stem Cells (DPSCs)	[23]
	Polycaprolactone (PCL)/gelatin methacryloyl	HUVECs/SMCs	[24]
	Gelatin methacrylate/catechol (GelMA/C)	Human Coronary Artery Smooth Muscle Cells (HCASMCs)/HUVECs/hMSCs	[25]
	Gelatin (or GelMA)/Alginate	HUVSMCs/HUVECs/HUASMC	[11]
Small caliber vessels (μm-scale)	Pluronic F127 (PF-127)/GelMA	Human dermal fibroblasts/HUVECs	[26]
	Polyethylene glycol diacrylate (PEGDA)	ECs	[27]
	Polyethylene glycol diacrylate	Lung cancer human alveolar basal epithelial cells (A549)	[28]
	GelMA/HAMA	HUVECs	[16]
	Glycine methacrylate-hyaluronic acid (GM-HA)/gelatinized methyl acrylate (GelMa)	Fibroblasts/HUVECs/HepG2	[29]
	Gel-NOR	HUVECs/MSCs	[30]
	GelMA/fibrinogen		[31]
	Methyl alginate acrylate (MeAlg)/MeHA/F127		[32]
Microvasculature (μm-scale)	Collagen/XG xanthan gum	hESC-ECs/fibroblast cell	[33]
	GelMA	HUVECs/HFFs/ADSCs	[34]
	Collagen	Neonatal Normal Human Epidermal Keratinocytes (NHEKs)/HUVECs	[35]
	Methyl acrylate/bone ECM	Human Dental Pulp Stem Cells (HDPSC)/Human Mesenchymal Stem Cells (HMSC)/HUVECs	[36]
	F127/gelatin/fibrin	Human Cerebral Microvascular Endothelial Cells (HCMEC)	[37]
	Silicate bioceramics Li–Mg–Si (LMS)/gelatin methacryloyl (GelMA)	ECs/nerve cells	[38]
	β-tricalcium phosphate (β-TCP) bone-derived dECM (BdECM)	human Adipose Stem Cells (hASCs)/HUVECs	[39]
	Pancreatic extracellular matrix (pECM)/hyaluronic acid	Pancreas cell	[40]
	Type I collagen sponges (col-1)/cartilage decellularized extracellular matrix (CdECM)	HUVECs/MSCs	[41]
	Gelatin-methacrylate/fibrin-based matrix	HUVEC/neuroblastoma cell/ADSCs/Human iPS cells	[42]

### 2.1 Biomaterials used for supporting vascular structures

According to the sources, biomaterials can be divided into two types, natural-derived biomaterials and synthetic biomaterials. Extracted from organisms, natural biomaterial can be classified into polysaccharide-based and protein-based. The characteristics of biomaterials are instrumental in governing vascular morphogenesis and the formation of capillary networks [43]. In comparison to natural biomaterials, which are constrained by their rheological and mechanical properties, synthetic biomaterials offer greater control over their physical attributes and bio-chemical potentials. This makes them particularly well-suited for the construction of mechanically robust vascular structures through a controlled manner. For more in-depth information on biomaterials used in bioprinting vascular tissues, please refer to Ref. [44].

The biological properties of biomaterials play a pivotal role in promoting vascular regeneration, with the process primarily influenced by components of the extracellular matrix (ECM) [44]. Matrices derived from ECM can either promote or inhibit the formation of capillary networks, thereby opening up new doors for the biofabrication of microvascular structures with anisotropy [41]. Therefore, studying the constituents and breakdown products of ECM is of great significance for controlling the formation of capillary networks. For instance, hyaluronic acid-ECM hydrogels and gelatin-norbornene (Gel-NOR) facilitates the attachment and growth of neointima, resulting in increased neointima density [40,45]. Furthermore, Collagen I has a positive impact on the condensation of endothelial cells (ECs) into extended geometries [46]. In summary, accounting for the biological properties of biomaterials is essential for successfully fabricating vascular structures and microvascularized structures.

### 2.2 Vascular-associated cells

ECs, Smooth Muscle Cells (SMCs) and pericytes are the basic cell types that make up the three-layer structure of the vasculature [47]. The commonly used ECs are human umbilical cord vein endothelial cells (HUVECs) [16] or human dermal microvascular endothelial cells (HDMECs) [21]. SMCs in the intermediate layer such as human umbilical vein smooth muscle cells (HUVSMC) [48], have high plasticity and also play a role in vasoconstriction, cell proliferation and ECM synthesis. The outer layer is majorly composed with pericytes which are involved in the production of ECM molecules and wound healing. For details on vascular-associated cells, please refer to Refs. [[49], [50], [51], [52]].

#### 2.2.1 ECs

ECs play key roles in physiology, including functions as a dynamic barrier, regulating blood flow and permeability, modulating vascular tone, and preventing thrombosis [53,54]. The vascular endothelium exhibits significant phenotypic and functional heterogeneity due to the varying degrees of stress and shear to which ECs are subjected [43]. ECs can be continuous, fenestrated or discontinuous, depending on their resident organizations [10]. Non-fenestrated continuous endothelium is present in arteries, veins, and capillaries of the brain, skin, heart, and lungs. It has selective properties, e.g., having a tightly connected blood-brain barrier (BBB) for vascular permeabilities [55]. Fenestrated endothelium occurs at sites of increased filtration or transendothelial transport, including gastric and intestinal mucosa, glomeruli, and renal tubules [5,9,55]. Discontinuous ECs have large openings that control the exchange of fluids, solutes, and macromolecules between the tissues and interstitial spaces [56,57].

However, bioprinted vascular structures have not been able to achieve cellular heterogeneity [49]. HUVECs are harvested from primary tissues (e.g., veins), which are used as the most common cells for constructing vascular constructs [25]. Indeed, fetal immune-privileged cell sources should be taken into account [58], and ECs phenotypes vary from one tissue to another [59]. Therefore, to recapitulate the functions in vascular tissue engineering, the sources of the cells that make up bioprinted vascular-associated cells should be matched to the specific tissues/organs.

ECs are the central and active part of the immune and vascular systems, which are important for angiogenesis and the stabilization of vascular systems [49,60,61]. After the vessel receives a generative stimulus signal, ECs are activated to become tip or stalk cells under the influence of angiogenic signals, migrating in through the gradient of GFs from the pre-existing vessels to non-vascularized areas [60]. In addition, ECs inhibit the entry of vascular mesangial cells into the endothelial layer during growth [49,61]. Furthermore, it has been confirmed that late-stage endothelial progenitor cells (EPCs) have the potential to clonally expand and to produce progeny, and such cells are sometimes referred to as endothelial colony forming cells (ECFCs) [49,62].

#### 2.2.2 SMCs

SMCs have two phenotypes, the post-differentiation quiescent phenotype and the post-dedifferentiation proliferative phenotype, which are associated with angiogenesis during the embryonic stage and with vascular repair during the stable phase of angiogenesis [[63], [64], [65]]. They are enveloped by a basement membrane, which function to inhibit the proliferation and migration of these cells. It is arranged so that SMCs are in a contracted state [66]. This basal lamina comprises closely interwoven collagen III fibers, and the diameter of the vessel significantly influences the thickness and abundance of these collagen bundles [67]. Additionally, SMCs play a crucial role in enhancing the mechanical stability of the vessel wall and regulating vascular tone [10]. For vascular scaffolds, the infiltration of smooth muscle cells (SMCs) is a critical factor for achieving functional neovascularization during regeneration [68]. To sum up, the precise control of the arrangement, the distribution and the physiological functions (e.g. proliferation, migration and infiltration) of SMCs, is critical when bioprinting vascular structures.

#### 2.2.3 Pericytes

Pericytes present in the capillary wall and between the small post-capillary veins facilitate angiogenesis. They are able to position themselves before the endothelial growth by determining the location of vascular bud formation, thus inducing the formation of new vessels [66,67]. The pericytes can switch between the proliferative stage and the differentiative stage, depending on the influence of several GFs, such as PDGF-B (Platelet Derived Growth Factor-B), TGF-β1 (Transforming Growth Factor-β1), VEGF (Vascular Endothelial Growth Factor) and Angs (Angiopoietin) [68]. It takes various roles in multiple biological processes, such as angiogenesis, BBB maintenance, immune cell regulation, and cerebral blood flow controls [69]. Additionally, pericytes make an important contribution to the formation and the integrity of the vessel walls in the microcirculation. It promotes ECs’ connections and facilitates the deposition of ECM components within the vascular basement membrane [68].

#### 2.2.4 Two types of cells for bioprinting vascular tissues

Vascular cells for tissue engineering are derived from different types of adult stem cells and progenitor cells, which are isolated from different sources, including bone marrow, adipose tissue, hair follicles, and umbilical cords [70]. Stem cells that can be potentially used for 3D bioprinting vessels are mainly human pluripotent stem cells (hPSCs), including human induced PSCs (hiPSCs) and human embryonic stem cells (hESCs). hiPSCs are derived to produce either arterial or venous-like ECs, both of which have the functional properties of somatic ECs [71]. In the study of Valeria V Orlova et al. [72], xenograft models made from hiPSC-derived ECs were able to integrate into the host vascular system, suggesting that hiPSCs have great potential for bioprinting of vessels. Furthermore, ECs derived from ESCs injected into the body can enter the peripheral ischemic sites, integrate into the microvasculature, increase capillary density, and further improve limb perfusion [73].

Endothelial colony-forming cells (ECFC) are subtypes of endothelial progenitor cells that can be isolated from umbilical cord or peripheral blood and have regenerative potential in the autologous environment [74]. They can be used to study hematologic diseases such as von Willebrand disease (VWD)/vascular hemophilia [75]. ECFCs can be derived from tissue-resident vascular endothelium, human umbilical cords or peripheral blood, or from human induced pluripotent stem cells [76]. It has been shown that ECFCs and mesenchymal progenitor cells (MPCs) can reconstruct blood flow in the ischemic tissues [77]. ECFCs can also be used for 3D bioprinting and can generate pre-vascularized tissue engineered structures [76].

### 2.3 Bioactive factors

The microenvironment composed of ECM contains GFs, differentiation factors, cell adhesion molecules, and components of ECM [78], which plays a crucial role in regulating cell fate and differentiation. The bioactive factors present in this microenvironment are vital for the in vitro cultivation of artificial blood vessels. Additionally, it is important to comprehend the mechanisms of natural angiogenesis aids in selecting suitable GFs to promote this process [79].

**Fig. 2:**
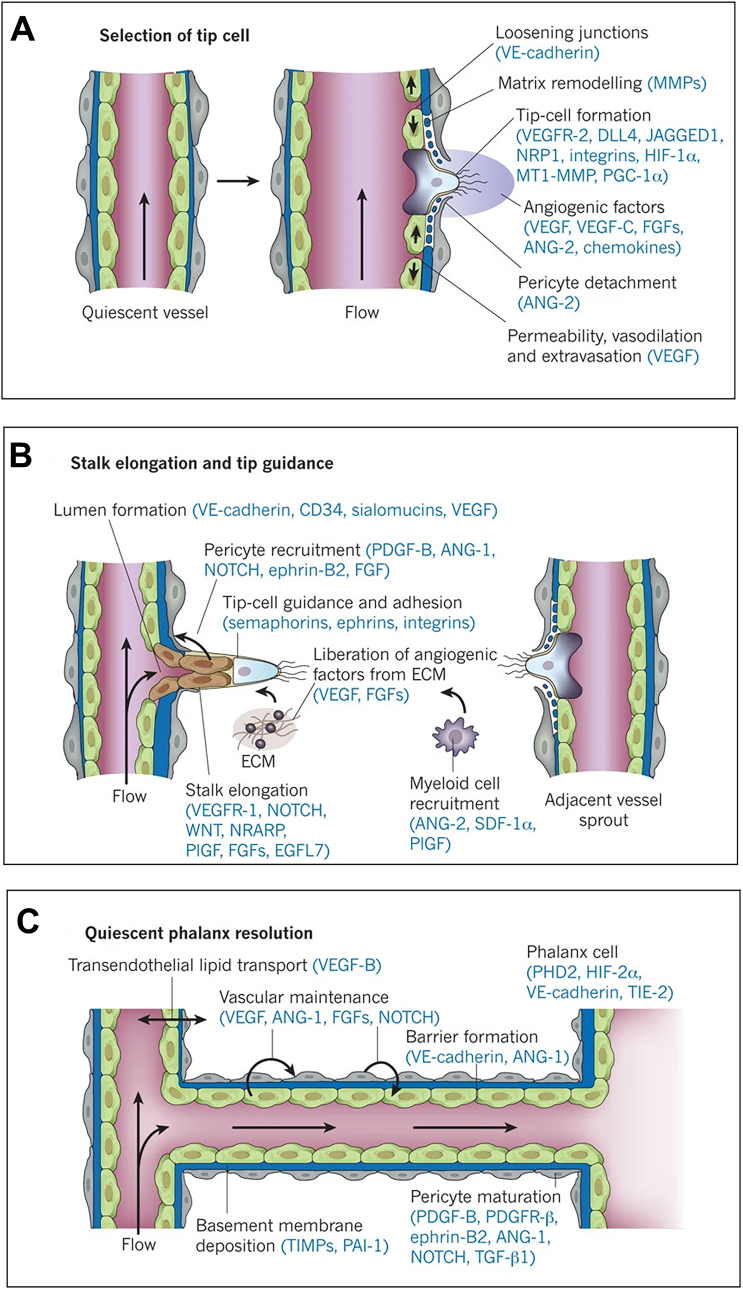
**Vascular branch formation steps.** Reproduced and adapted with permission [80]. Copyright 2011, Springer Nature. A) In response to stimulation with angiogenic factors, quiescent vessels dilate and a particular EC is selected as the tip cell. B) The stem cells after the tip cells form a lumen by proliferation and finally fuse with the adjacent vessels by budding to form a neovascularization. C) After fusion of adjacent vessels, the lumen allows neovascular flow and restoration of quiescence through a series of further complex processes, with the end of vessel branch formation.

The emergence of natural vessels is driven by hypoxic conditions, and generated in the following major steps (Fig. 2) [80]. Traditionally, the generation of vasculature is a process in which ECs residing in the periphery of the vessel branches outward from a local niche. A small number of circulating endothelial progenitor cells are directly involved in the formation of new vessels [81]. Currently, the mechanisms of natural vessels generation are generally divided into two categories: angiogenesis and vasculogenesis [80].

Hundreds of molecules and factors are involved during vasculature development, mainly including ECM components, integrins, chemokines and GFs [82]. Here, we have summarized several chief GFs: VEGF, PDGF, FGF (Fibroblast Growth Factor), TGF-β, and Ang, as described in the table below (Table 3). ECs are inoculated into multi-scale microfluidic channels to stimulate the formation of monolayer vascular endothelium. This is achieved by incorporating GFs into the constructs. An alternative approach involves pre-mixing ECs and co-cultures of other cell types specific to the target organ within the tissue scaffold beforehand. This strategy leverages the inherent biological properties of ECs to facilitate the formation of new capillaries in vivo [83]. Apart from natural GFs, bioactive ions can also indirectly contribute to the promotion of angiogenesis. A study showed that the ions of Li, Mg, and Si can promote the expression of angiogenic genes in a concentration-dependent manner [38]. This effect is primarily achieved by upregulating VEGF expression, initiating the angiogenic signaling pathway, and subsequently enhancing the angiogenic capacity of HUVECs.

**Table 3:** Functions of the GFs.

GFs	Functions	Ref.
VEGF	Angiogenic bud formation VEGF-A: transform EC into tip/stalk cells	[44,84,85]
PDGF	Recruiting pericytes and SMCs Stimulating vessel growth and maturation while also attenuating the response to anti-VEGF therapy	[44,80,86]
FGF	FGF-2: stimulating ECs barrier integrity bFGF: promoting migration, proliferation, and survival of ECs and SMCs	[[87], [88], [89], [90]]
TGF-β	Inhibiting ECs invasion and capillary lumen formation Limiting ECs apoptosis and block EC migration Stabilizing blood vessels	[44,82,91]
Ang	Ang1: promoting ECs survival and vascular stabilization and tightness Ang2: regulating angiogenesis and regression, promote pericyte separation and vascular permeability	[44,80,92,93]

## 3 Bioprinting technologies

To achieve functional structures in bioprinting, apart from selecting suitable biomaterials as alternatives to ECM and cellular sources, the three-dimensional structure defined through the fabrication method plays a crucial role [94]. The choice of bioprinting technique is closely related to the physical and chemical properties of the bioink. Meanwhile, the ideal biomaterial can be modified to construct structures with high resolution and fidelity in line with bioprinting principles. However, cell viability is primarily constrained by the bioprinting methods, mainly due to the physicochemical factors that can be harmful to the cells. For instance, photons in Laser-assisted bioprinting (LaBP), poorly degradable photocurable materials in stereolithography (SLA), shear forces in extrusion bioprinting, as well as mechanical stress and heat in inkjet bioprinting are all the possible influencing factors [95,96]. Above all contribute to a significant reduction in cell activity, ultimately impacting the culture and functional development of cell-containing structures.

**Fig. 3:**
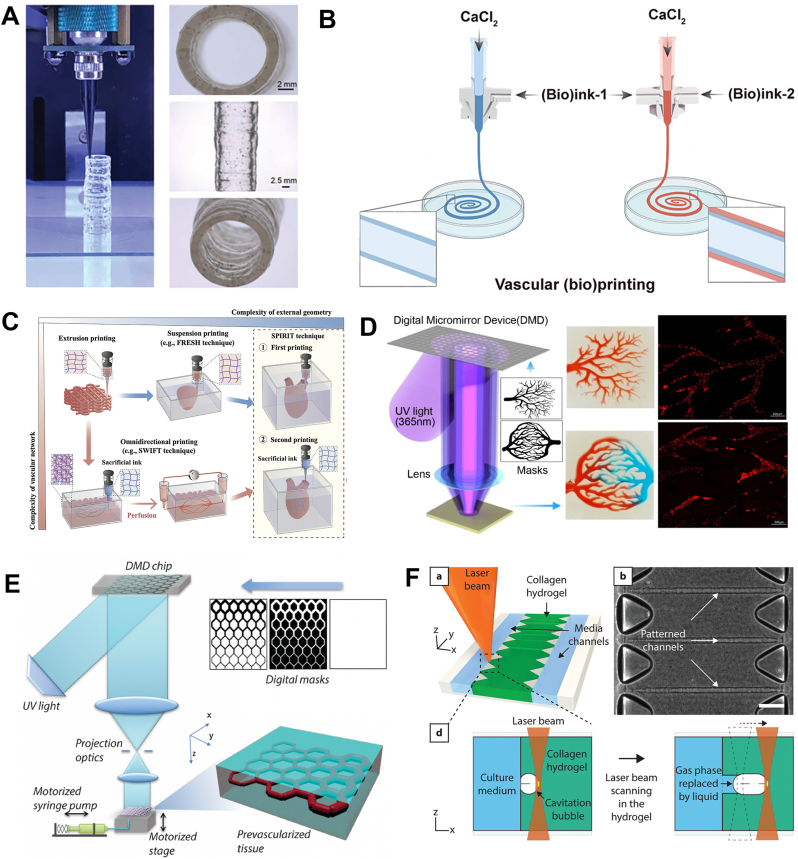
**Bioprinting techniques for fabricating vascular structures.** A) Extrusion-based bioprinting: extrusion of single-layer tubular structures. Reproduced and adapted with permission [19]. Copyright 2021, John Wiley and Sons. B) Schematic diagram of microfluidic extrusion bioprinting of single and bilayer vascular structure. Reproduced and adapted with permission [11]. Copyright 2022, American Association for the Advancement of Science. C) Sequential fabrication of tissue and organ structures with complex external geometry and vascular structure using microgel biphasic (MB) hydrogel bioink and sacrificial bioink. Reproduced and adapted with permission [98]. Copyright 2023, John Wiley and Sons. D) Projection-based 3D printing system to construct complex vascular network structures. Reproduced and adapted with permission [28]. Copyright 2018, American Chemical Society. E) Continuous 3D printing process using UV-LED induced photopolymerization of photosensitive biomaterials, individually controlled DMD with continuous input of a series of digital masks while moving the printing platform. Reproduced and adapted with permission [29]. Copyright 2017, Elsevier. F) In situ formation of vascularized tissue models using femtosecond laser irradiation of collagen hydrogels.Reproduced and adapted with permission [99]. Copyright 2022, John Wiley and Sons.

The bioink formulation plays a crucial role in determining the bioprinting approach, particularly in terms of cross-linking principles, rheological properties, and other physical characteristics. Extrusion bioprinting has become one of the most commonly utilized manufacturing techniques in recent years due to its ability to accurately deposit bioinks and create simple or complex vascular or prevascularized structures based on target designs (Fig. 3A) [19]. This popularity can be attributed to the favorable rheological properties of most bioinks. However, extrusion bioprinting is primarily suited for high-viscosity hydrogels [97], making it unsuitable for shaping low-viscosity liquid biomaterials. To address this problem, several bioprinting strategies have been proposed to fabricate vascular-like structures, with coaxial bioprinting and embedded bioprinting being the main approaches.

Hiroaki Onoe et al. [100] developed a coaxial microfluidic device based on the laminar flow principle and successfully produced hydrogel microfibers with a core-shell structure, capable of mimicking the morphology and functionality of living tissues. Coaxial bioprinting with axial nozzles offers the potential for creating hollow structures [101], including perfusable vascular networks containing cells [102]. Advancements in this technique have enabled the construction of single and multi-layered vascular structures. To enhance biological relevance, triple-layered vessels with both layers of ECs and smooth muscle cells can be formed by increasing the feed port and utilizing different cross-linking methods for bioinks. Di Wang et al. [11] cleverly employed dual network hydrogels to microfluidic print single/double-layered hollow conduits, resembling venous and arterial-like tissues, respectively (Fig. 3B). Although current coaxial bioprinting techniques faced difficulties in constructing capillaries and branching vascular structures, they held significant potential for fabricating hydrogel tubular structures with complex shapes that mimicked specific vascular microenvironments or disease models [103]. This is of great importance in studying cellular interactions and developing intricate tissue-like models [94].

To address the issue of soft hydrogels collapsing and failing to maintain their intended shape, Thomas J et al. [104] successfully printed a structure to mimic the branching coronary artery firstly using a gelatin particle suspension bath called FRESH. In this embedded-based bioprinting approach, the sacrificial material served as both a sacrificial bioink for creating molds of hollow structures or networks and as support for the bioprinting. In addition to the endothelium-containing bioink to form the channels during fabrication, ECs can be subsequently implanted in the conduit, leading to endothelialization [58].

While single or multi-layered vascular structures have been successfully constructed, reproducing tissue organs that include complex vascular structures remains a challenge. Yongcong Fan et al. [98] proposed the Sequential Printing In a Reversible Ink Template (SPIRIT) technique (Fig. 3C), which demonstrated the potential to create intricate tissues and organs with highly complex external shapes and internal structures. The support bath was used to form complex external features like ventricles; while the sacrificial bioink was printed within the uncross-linked structures. This process enabled the formation of freeform vascular networks through self-healing bioink. By incorporating a self-healing and biodegradable hydrogel as the support bath, it became possible to construct complex geometries, including linear or branching vessels. This bioprinting strategy offers the capability to create intricate and specific vascular shapes.

Extrusion-based bioprinting has certain limitations [97]. Firstly, the height of each printed layer is fixed, which also restricts the height of the sacrificial ink. Increasing the height of the sacrificial ink alone may result in lumen obstruction upon removal. Additionally, many studies utilized hydrogels as the primary material, which can degrade over time, leading to lumen collapse. Furthermore, bioprinting bifurcated vessels and small diameter capillary networks currently faces many challenges. As a result, higher precision bioprinting methods have been proposed. Vat polymerization (VP)-based bioprinting represents an emerging bioprinting technology encompassing SLA, digital light processing (DLP), and two-photon polymerization (2 PP) [105]. All of these techniques are under the category of Light-Assisted Bioprinting. One study employed two-photon laser scanning photolithography to replicate the intricate microenvironments of vascular ecological niches [106]. Additionally, there is the innovative technique of volumetric printing (VP), a recent development in the realm of biofabrication, which is also in the domain of light-based volumetric bioprinting [107]. Drawing inspiration from the principles of optical tomography relying on visible light projection [108], this technology bestows a higher degree of design flexibility compared to traditional bioprinting methods [24]. Notably, it enables the swift fabrication of tissue structures within a concise time, showcasing substantial promise for the creation of vascular tissues [109].

Stereolithography-based bioprinting transforms light-sensitive liquid materials into solid structures using laser or projection methods [96]. This technique allows for the incorporation of various biomaterials, cells, and biomolecules into the printed tissue structures [39]. One particular approach, projection-based stereolithography, can cross-link photocurable materials in a single pass based on digital images, reducing fabrication time compared to other bioprinting techniques [44]. Stereolithography promotes rapid biomanufacturing, maintains the viability of cells and supports the normal function and differentiation of stem cells [27]. Using this technique it is possible to construct a lot of structures from regular geometries to more complex geometries, such as bionic tree shapes and capillary networks(Fig. 3D) [28]. However, it does have certain limitations for which several proposed solutions can be considered. Firstly, the patterns formed by encapsulating cells in hydrogels can be combined with cell seeding after fabrications to enable culture patterns that mimic natural tissues and organs. Secondly, vascular occlusion can be reduced through some surface treatments of the scaffold. Lastly, changing the variable resolution of printing allows for the fabrication of large scaffolds with complex microstructures, expanding the applicability of stereolithography in tissue engineering applications.

In contrast to stereolithography, which operates on a one-dimensional level, DLP based bioprinting functions on a two-dimensional level. Using digital micromirror array devices (DMDs), DLP enables the controlled 3D fabrication of complex structures (Fig. 3E) [29]. Levato et al. utilized a novel light-responsive bioresin to fabricate brain vascular anatomical 3D with an out-of-plane branching network, paving the way for bioprinting complex channel structures [110].

In addition, LaBP enables the easy fabrication of perfusable capillary-scale networks without sacrificing materials [35,111]. To address issues such as photothermal processes that can impair cell viability and hydrogel integrity, in situ 3D imaging of collagen hydrogels using femtosecond laser irradiation has been reported in the research to create channels and cavities ranging from 20 to 60 μm in diameter (Fig. 3F) [99]. Although the biological structures created using LaBP technology are not fully functional, it allows for the printing of more complex and highly active models with high precision compared to other bioprinting methods.

As bioprinting technology continues to advance and evolve, it has achieved remarkable progress in the field of vascular tissue engineering. In addition to bioprinting, there are several traditional methods of vascular tissue engineering (VTE) (Table 4). Back in 1912, Carrel pioneered the use of silicone rubber to craft artificial arteries and successfully fused them with native blood vessels [109]. Subsequently, vascular engineering techniques have undergone continuous development. Conventional strategies for vascular fabrication encompass electrospinning (ES) [112], cell sheet-based techniques [34], decellularized tissue-based approaches [113], and freeze-drying methods [114]. In contrast to the creation of centimeter(cm)-scale vascular structures, laser-based hydrogel degradation technique is frequently employed to generate μm-scale vascular networks [53]. Additionally, external physical stimulation methods have been instrumental in promoting the generation of capillary networks, such as sound-induced methods [115] and electrical stimulation methods [116]. For an in-depth review of fabrication methods in VTE, please refer to Refs. [5,117]. Notably, there is a study on constructing coronary, arteries using electrostatic spinning and freeze-drying methods [114], which suggests that the fusion of multiple techniques might be a promising approach.

**Table 4:** Manufacturing strategies for vascular tissue engineering.

Vascular engineering techniques		Classification of cailber vessels	Applications/Utilities	Ref.
3D bioprinting	Droplet-Based Bioprinting	Vascular structure	Potential to be used as clinical grafts	[18,118]
	Rotary Bioprinting	Small-diameter vascular constructs		[[119], [120], [121]]
	Extrusion-Based Bioprinting	Vascular scaffolds	Vascular stents with good mechanical properties/disease models	[14,19,21,22,32,122]
	Embedded Bioprinting	Multi-level vascular network structure	Reproducing the structural and functional properties of natural tissues	[15,31,98]
	Coaxial Bioprinting	Constructs with vascular channels/veins and arterioid tissues	Generating drug screening models/vascular models for disease research	[11,20,94,[123], [124], [125]]
	Laser-assisted Bioprinting (LaBP)	Vascular network	3D Tissue models with complex microvessel	[35] [99,126]
	Digital light processing (DLP) based Bioprinting	Vascularized Constructs	Anastomosis between the grafted prevascularized tissues and the host vasculature/drug testing models	[29,30,36]
	Light-Assisted Bioprinting	Vascular structure	Microvascular organ model studying the physiological function of blood vessels	[16,17,27,28,127]
	Volumetric Bioprinting	Complex structure with a branched channel	Simulation of early angiogenesis/complex heterogeneity of living tissues	[24,108,128,129]
Traditional manufacturing strategies	Micro-needles (the “Kenzan” method)	Tubular structure	Scaffold-free biofabrication	[[130], [131], [132]]
	Sound-induced Methods	Vascular networks	Tumor/drug screening model	[115,133,134]
	Electrical stimulation			[116]
	Electrospinning (ES)	Well-designed vascular graft	Ideal candidates for vascular graft application	[112,[135], [136], [137]]
	Cell sheet-based	Small diameter arteries/vessels	Recreating the hierarchical structure of vessels	[3,4,8]
	Decellularized tissue-based	Decellularized vascular grafts	Potential for clinical translation	[113,138,139]
	Freeze drying methods	Bilayer heparinized vascular graft		[114]
	Dual phase separation technique	Macroporous nanofibrous/vascular scaffold		[140]
	Dipspinning methodology	Vascular grafts of human coronary arteries		[141]
	Laser-based hydrogel degradation technique (photodegradation)	Vascular networks	Fabricating advanced on-a-chip devices with high-density microfluidic networks	[53,54]

In a similar vein, the advantages of different bioprinting techniques were combined to fabricate vascular tissues with different scale. A recent study combined coaxial bioprinting and stereolithographic bioprinting to construct vascularized tissues [25]. Moreover, methods like DLP based bioprinting combined with extrusion bioprinting [30], Embedded Extrusion-Volumetric Printing (EmVP) [142], and embedded bioprinting combined with extrusion bioprinting [98] were developed for creating vascular structures. Notably, bioprinting can also be integrated with traditional techniques to replicate mechanically robust functional vessels. The combination of volumetric bioprinting and melt electrowriting (MEW) was cleverly employed to produce engineered tubular structures [24]. In conclusion, the integration of these multiple techniques presents novel research avenues for crafting mechanically adaptable, multilayered vascular structures, and tissues featuring intricate vascular networks.

## 4 In vitro assessment of vascular cell functions

To effectively create functional vascular tissues, multiple approaches have been devised, along with the development of various bioprinting techniques and their corresponding bioinks. The manufacturing solutions for different levels of vascular structures vary based on their intended functional and usage. Currently, in the field of bioprinting, there are three primary application scenarios for artificial vessels: (1) Large-diameter vessels for graft replacement; (2) Endothelialized hollow channel structures that mimic medium-scale natural vessels; (3) Capillary networks formed through spontaneous induction.

Numerous bioprinted vessels have been fabricated and the related studies have demonstrated their ability to perform physiological functions. However, many of these studies have focused primarily on achieving structural resemblance without attaining true consistency in both structure and function. In order to further understand the physiological functions of artificial vessels, we systematically described the current criteria for assessing vascular tissues as well as their functions.

The long-term survival of cells in tissue-engineered vascular substitutes remains one of the most topical concerns in the field of biomanufacturing [143]. It is also one of the main indicators for the function of bioprinted vessels. The optimization of bioink formulations and the continuous innovation of bioprinting methods have made it possible to manufacture complex tissues or organs. However, the cell survival after bioprinting is one of the key factors to obtain functional structures in further culture; while maintaining high cell viability is extremely challenging. In addition, cell viability can be measured to verify the interactions between biomaterials and cells [144]. It consequently has an impact on several typical cellular behaviors, such as cell division, migration and death. Specifically, the morphology and the survival of cells can be observed by characterizing specific proteins on the surface of different cell types or inside of them. The process of vascular function such as ECs extension, migration, and angiogenic sprouting can also be traced in vitro. Some common indicators of vascular-associated cell function are listed in Table 5. It is worth noting that factors in the environment of cell growth play an important role in the performance of vascular tissue function.

**Table 5:** Vascular tissue function: assessment of vascular-associated cell.

Vascular-associated cell	Initial cell survival rate (%)	Characterized proteins	Factors affecting cell survival	Ref.
HUVECs	>70	CD31 VE-cadherin	Dynamic cultivation Increased cell density	[145,146]
	90	No data	Adhesive peptides in bioink	[147]
Fibroblasts	60–70	No data	Shear force	[148]
MSCs/HUVECs	80	α-SMA CD31		[102]
A549	100	No data	Cell seeding density Friction	[149]
HUVECs/cardiomyocytes	No data	CD31 Connexin-43	Oxygen throughput Culture fluid Perfusion flow rate	[150]
hMSCs	95	CD-31 vWF VE-Cadherin	Distance of the surrounding cells from vessels	[151]
HUVECs/HUASMC	83	CD31 SMA VE-cadherin Type IV collagen		[152]
			Culture methods	

### 4.1 High cell viability as a prerequisite for vascular functions

The diffusion limit of oxygen and nutrients in nonvascular tissues is generally 100–200 μm, which can significantly affect regular cellular behaviors such as cell division, migration, and death [144,145]. Hence, cell viability is one of the most important indicators for vascular tissue function. However, cell viability is influenced by many factors during the bioprinting process, including the seeding methods of the vascular-associated cells, the cell seeding densities, and the mechanical forces to which the cells are subjected.

The inner layer of natural vessels consists of uniformly dispersed vascular ECs [153]. To simulate a monolayer vascular structure, indirect bioprinting or coaxial bioprinting is usually used to construct hollow structures [145,150,154]. These cells are then perfused into the channels with a suspension of ECs. After a period of cultivation, these cells firstly become a basically flat, partially cobblestone-like organization, and ultimately form a vascular structure containing a single layer of ECs. When using this conventional cell seeding technique, cells within the channel are unevenly distributed. The adherent cells distal to the point of perfusion are significantly reduced, and thus their cell viability is also reduced. In order to solve the above problem, Ouyang et al. [153] came up with an in situ seeding method (i.e., void-free 3D printing approach) in which bioink mixed with ECs was used as a sacrificial material. The liquefied bioink was retained in the channel after bioprinting, resulting in uniform cell dispersion in the vessel wall. Although the diameter of this vessel did not strictly match with the size of natural vessels [155], this hollow structure with well-distributed cells mimicked the geometry of natural blood vessels and can transport nutrients and oxygen.

The method of seeding cells after bioprinting was used to construct monolayer vessel with uniform cell distribution, which has strict requirements on the density, viability, and flow rate of cells. Xue et al. [149] constructed a multi-level branching network structure to study cell metabolism and nutrient transport. However, the unevenness of the cell suspension resulted in cell clogging at the branches, which lowered cell survival. Large friction between the cells and the tube wall was avoided by reducing the initial cell seeding density, which significantly boosted the cell survival rate. Additionally, some studies had noted a decline in initial cell survival, which may be related to the shear or tensile stresses that cells were subjected to during the bioprinting process [148].

The approaches to measure cell viability include the detection of metabolic activity, cell proliferation, cell membrane leakage, and dynamic cellular responses to external stimuli [156]. Especially, the distribution of dead and live cells in perivascular tissues can be observed with the help of dead/live cell staining. Previous study showed that cell viability rapidly decreased with the increase of the cell density beyond 1 mm from the vessels by assessing the viability of the cells in and around vessels in thick vascular tissues [151]. In another study, vascular channels were constructed among HepG2 spheroids, and the size of the spheroids increased as the distance from the channels decreased [157]. It can be concluded that cells close to the channels underwent rapid proliferation; while cells at distant area behaved essentially death or no cell division. Thus the distance between vessels was critical for the survival of the perivascular cells. Additionally, the viability of vascular-associated cells and perivascular cells in turn influenced the ultimate tissue functions.

### 4.2 Specific markers to characterize the vascular cells

**Fig. 4:**
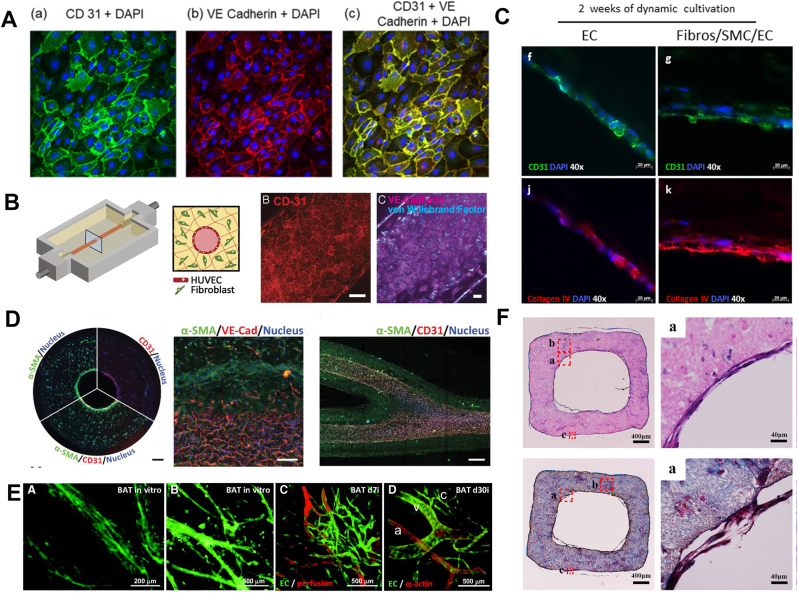
**Characterization and use of vascular-associated cellular markers.** A)-B) The marker CD31/VE-cadherin/vWF shows cellular junctions between monolayers of ECs. Reproduced and adapted with permission [158]. Copyright 2020, Springer Nature. Reproduced and adapted with permission [164]. Copyright 2016, Proceedings of the National Academy of Sciences. C) The vascular structure is composed of three layers of cells in which collagen IV represents the microvascular basement membrane Reproduced and adapted with permission [152]. Copyright 2018, Springer Nature. D) Confocal images of bifurcated vascular structures after 10 days of perfusion culture were obtained, and they were labeled with CD31, α-SMA, and VE-cadherin to visualize the fusion layer of HUVECs. Reproduced and adapted with permission [103]. Copyright 2022, John Wiley and Sons. E) Classification of vessels by determining the amount of α-actin. Reproduced and adapted with permission [165]. Copyright 2011, Wolters Kluwer Health, Inc. F)The H&E staining and Masson staining of bionic vascular vessel. Reproduced and adapted with permission [17].Copyright 2022, John Wiley and Sons.

Immunohistochemistry is commonly used to assess the vascular phenotype at the cellular level, to quantify vessel diameter and vessel density, and to visualize the degree of vascularization. The secreted proteins used to characterize ECs are currently limited. Since the most classical cellular signature proteins are cellular structural proteins, it is reasonable to using several important endothelial-specific membrane structural proteins, including CD31 and Vascular Endothelial Cadherin (VE-cadherin) to characterize ECs (Fig. 4A) [158]. CD31, also known as a platelet-endothelial cell adhesion molecule, has vital roles in mechanotransduction, metabolism and immune function [159]. VE-cadherin is an endothelial-specific adhesion molecule located at the junctions between ECs. Both of them are also pro-angiogenic and cell-adhesive proteins [160]. Meanwhile, the von Willebrand factor (vWF) has been reported in some literature to characterize ECs (Fig. 4B) [151,[161], [162], [163]]. Although vWF is mainly produced by them, it is not the preferred method for the characterization of ECs because it is produced in high volumes during hemostasis and megakaryocytes can also secrete the modest amounts of vWF. In addition, type IV collagen, the main component at the borders between the basal lamina and the proximal luminal surface of the endothelium, can also serve as a marker for ECs (Fig. 4C) [152]. Therefore, CD31, VE-cadherin and type IV collagen are commonly used to characterize ECs.

Pericytes are cells that exist at intervals along the capillary walls [166]. Unlike other vasculature-forming cells, pericytes differ not only in morphology but also in protein expression among diverse organs at different developmental stages. It specifically expresses proteins such as smooth muscle α-actin (α-SMA) and proteoglycan Neuron-glial antigen 2 (NG2) [167]. Among them, α-SMA is also a signature protein of SMCs (Fig. 4D) [103]. During vascular remodeling and repair, pericytes differentiate into ECs and SMCs [168]. Since the phenotype of pericytes has not been strictly defined, the differentiation and the development of them have not been comprehensively studied. Besides, it has been found that hADSCs also played a pericyte-like functional role during vascular development, and a tight co-localization of pericytes with HUVECs was observed using NG2 as a molecular marker [169].

In summary, the characterization of specific proteins or ECM proteins provides the insight into the state of existence as well as the function of vascular component cells at the cellular level. In addition, it is also possible to identify vessels according to the distribution and the organization of these signature proteins. One study recognized vessels based on the arrangement of α-actin (Fig. 4E) [165]: (1) Tiny arteries, thick banded α-actin band (smooth muscle) coverage; (2) Small veins, smaller smooth muscle or non-small arterial smooth muscle coverage; (3) Capillaries, no smooth muscle at all. Additionally, H&E staining and Matson trichrome staining (MTS) are frequently employed at the histological level to more accurately visualize in vitro vessel formation [39]. It clearly shows the shape of the vessels and the distribution of the cells, which facilitates the observation of their structural integrity (Fig. 4F) [17].

### 4.3 External environmental factors affecting the vascular functions

In tissue-engineered structures, ECs are the major cell type for the formation of blood vessels. The interaction between ECs and perfusion fluids (such as blood) or culture fluid affects the development of blood vessels [117]. When a fluid goes through a vessel, the cells are generally subjected to the circumferential and shear stresses (acting tangentially and longitudinally on the vessel wall), respectively. The cells sense the mechanical signals provided by the microenvironment [143]. These mechanical forces activate a complex cascade of cellular signaling pathways, leading to the alterations in the intracellular functions [170]. In the natural vasculature, hemodynamics plays a major role in mediating mechanical forces; while the synthetic vessels are cultivated in a specially designed bioreactor in which fluid sends mechanical signals to the cells. However, the culture conditions for the development of EC layers and the budding of capillary call for different mechanical stresses. The former is aided by the flow situation; while the latter can also benefit from the static culture [171].

Mechanical factors contribute to angiogenesis, particularly shear stress which promotes the upregulation of VEGF expression [8]. Under conditions of uniform shear stress distribution, there is a favorable environment for ECs maturation and endothelialization of channels [2,172]. Moreover, flow kinetic characteristics (e.g., pressure, axial velocity, and shear rate) play a crucial role in the physiological behavior and function of cells on the channel surface under in vitro conditions. Interestingly, electrical stimulation (ES) has been found to significantly promote the elongation, migration, and interconnection of HUVECs, as well as increase the expression of relevant genes. Additionally, the stiffness of the biological scaffold and the signals (e.g., oxygen gradients, cell adhesion ligands, and the types of GFs [58]) in the microenvironment are all critical to the sprouting of ECs. Mechanosensors on the cell membrane can sense the stiffness of the scaffolds and perform a role in cell migration and growth. ECs on a soft matrix formed a partially fused cobblestone-like layer; while cells on a stiff matrix would generate a lamellar structure [45]. In a word, the selection of appropriate physical stimulation has a positive impact on angiogenesis.

Co-culture systems play a pivotal role in stabilizing neovascular networks and enhancing the functionality of vascular structures [58]. Specifically, the interaction between vascular ECs and surrounding cells influences the development of vascular tissues. Tânia Baltazar et al. [173] demonstrated the production of vascularized skin tissues, wherein ECs and pericytes facilitated the maturation of human foreskin keratinocytes (KCs) and self-organized into interconnected vascular networks. Furthermore, research has also shown that co-culturing ECs with stem cells enhanced the vascularization of bioprinted structures [30].

Notably, the sustained effects of paracrine-associated growth factors, secreted by nonvascular cells, significantly contribute to vessel formation. Paracrine signals, such as VEGF produced by osteoblasts or Mesenchymal stem cells (MSCs), induced angiogenesis in surrounding ECs [174]. Similarly, the paracrine signals provided by MSCs in co-cultures of ECFCs and MSCs promoted the formation of capillary networks driven by ECFCs [108]. The upregulation of paracrine signaling in the co-culture system of endothelialized hMSC/HUVEC accelerated osteogenic differentiation [129]. Wang et al. discovered that glioma cell U118 exhibited a significantly enhanced ability to secrete bFGF and VEGF, which promoted vascularization in a vascularized tumor model [175]. Considering the hierarchical diversity of the vascular system, as well as the role of different tissues in relation to the vasculature [58], the types of cells incorporated into a co-culture model also exert a substantial influence on the ultimate function of the vascular tissue. The overarching objective is to integrate various cell types into the co-culture system to promote the formation and functional stability of vascular tissues.

Achieving a cellular arrangement similar to that found in natural organs through bioprinting remains a formidable challenge. Strategies involving the co-culturing of vascular constituent cells and tissue cells showed promise in partially emulating multi-cellular interactions in vivo [21]. The currently bioprinted vascular structures, which contain ECs, SMCs, and fibroblasts, are inadequate for the development of fully functional vessels. It is imperative to consider factors within the cell culture environment, such as fluid perfusion, scaffold stiffness, and GFs. Addressing these elements can potentially lead to the maturation of vessels and the fulfillment of their ultimate functions in vitro.

## 5 Assessment of vascular tissue functions

**Fig. 5:**
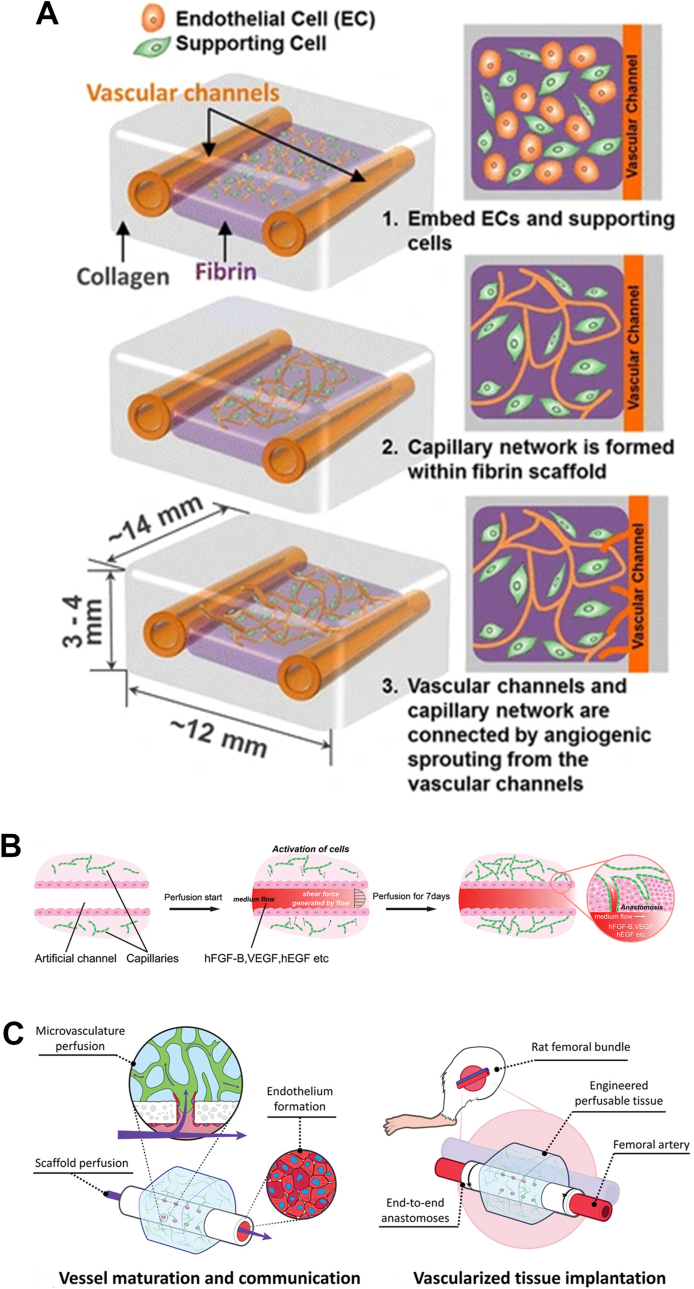
**Interaction, growth and anastomosis between large diameter vessels and the capillary network of the surrounding tissue.** A) A multi-scale vascular system manufactured using 3D bioprinting technology simulates the process of vessel growth and maturation. Reproduced and adapted with permission [171]. Copyright 2014, Springer Nature. B) Mechanical signals generated by perfusion culture, along with chemical signals such as GFs, synergistically enhance capillary-channel anastomosis. Reproduced and adapted with permission [8]. Copyright 2023, John Wiley and Sons. C) Hydrogels spontaneously form vessels and get attached to tubular scaffolds, allowing the microvascular system to be perfused. Reproduced and adapted with permission [176]. Copyright 2021, John Wiley and Sons.

Currently, the following challenges exist in creating fully functional vascular tissues in vitro [144]: (1) the fabrication of functional multilayer large/small diameter vessels; (2) the fabrication of large-scale vascularized tissues; (3) integration of vascular (vascularized) structural grafts with their own vessels or natural blood vessels. The anastomosis of capillaries and channels makes it possible to deliver oxygen and nutrients to the interior of the tissues. Vivian K. Lee et al. [171] constructed a vascular thick tissue model that combined a capillary network embedded in a hydrogel with a single layer of large diameter vascular channels (Fig. 5A). Due to the slow generation rate of vascularized tissues prepared by bottom-up methods, strategies to fabricate vascular tissues using top-down approaches have been proposed. Tubular structures assembled from vascularized cell sheets were capable of forming hierarchical vascular networks through vascular anastomoses (Fig. 5B) [8]. This result paved the way for the construction of high cell density tissues containing large vascular structures and vascular networks. For instance, Szklanny et al. [176] assembled the micro-vascularized hydrogels with the vascular scaffolds to achieve Host-To-Implant perfusion recently (Fig. 5C). In addition, the features such as perfusability, permeability and mechanical strength of vascularized tissues should also be taken into account **(**Table 7**)**.

**Table 6:** A summary of bioreactors for culturing bioprinted vascular tissue.

Biofabrication structure	Culture method	Composition of bioreactor	Culture time	Ref.
Endothelialized-myocardium-on-a-chip	Microfluidic bioreactor	Two hemi-chambers/Teflon microtubes/silicone tubing/a peristaltic pump	2 weeks	[177]
Vascular channel	Dynamically cultivate	A peristaltic pump/silicon tubes in 37 °C and 5 % CO2.	2 weeks	[18]
Scaffold-free engineered tissue construct	A perfusion bioreactor	No	No date	[178]
Vascular channel construction	Flow chamber and perfusion System	Flow chamber/perfusion system/silicon tube/polypropylene fittings	5 days	[118]
Vascularized construct	Dynamic culture	A media reservoir/a flow-chamber/a peristaltic pump/silicone tube	3 weeks	[179]
Branched vascular networks	A continuous flow perfusion system	An incubator/a peristaltic pump/a reservoir of culture medium	1 week	[31]
Perfusable hydrogel construct	Perfusable culture	A peristaltic pump/the hydrogel microfluidic device	2 weeks	[180]
Vascularized tissue structure	Dynamic culture	Silicone bioreactor/Perfusion fluidics setup	1 week	[37]
Small-diameter vasculature		PDMS chamber/a digital peristaltic pump/a fluid reservoir	2 weeks	[25]

**Table 7:** Assessment of vascular tissue function.

Vascular structure	Permeability/Perfusability	Mechanical strength	Vascular anastomosis	Ref.
Arteriole-like trilaminar structure	No data	Modulus of elasticity close to that of a natural aorta	No data	[181]
Hollow grid structure	Transplantable Structural integrity	No obvious signs of collapse or breakage	No data	[102]
Bending pipe construction	Break highly permeable without clog	Withstand tensile and compressive forces in a short time	No data	[182]
Prevascularized spheroids	No data	No data	Capillary anastomosis between cellular spheroids	[183]
Thick tissue with hollow channels	Permeable	No data	No data	[151]
Honeycomb grid structure	Perfusability	No data	Vascular anastomosis with host epithelial tissue	[29]
Tubular structure		Multi-layer pipes are stretchable and deformable	No data	[184]
Liver tissue containing multi-level tubular structures model	Barrier function	No data	Direct anastomosis with the host vessel	[146]

### 5.1 Vascular permeability

Generally, nutrients permeate through the intima to the underlying tissues [185]. Therefore, the vascular permeability is quite important to support the function of nutrient transportation for blood vessels. It is generally determined by the features such as the continuity of the endothelium and the presence or absence of pores. For instance, the renal filtration barrier is made up of a discontinuous and highly permeable endothelial monolayer; while BBB is created by a layer of continuous and nonporous ECs [186]. However, the bioprinted vessels were relatively simple and had the fundamental permeability.

**Fig. 6:**
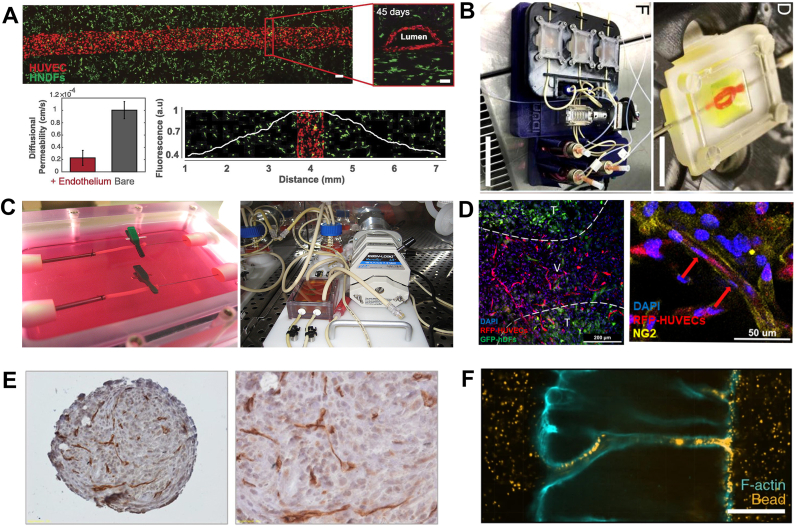
**Vascular tissue with perfusability and perfusability.** A) The vascular network achieved long-term perfusion. The endothelium provided a screen of barrier properties and permeabilities. Reproduced and adapted with permission [151]. Copyright 2016, Proceedings of the National Academy of Sciences. B)–C) Large diameter tubular structures were perfused in a specially designed fluid platform for processing. Reproduced and adapted with permission [157]. Copyright 2018, Elsevier. Reproduced and adapted with permission [189]. Copyright 2009, Elsevier. D) Pericytes-ECs co-localization and generation of new vessels at the tissue interface. Reproduced and adapted with permission [169]. Copyright 2012, American Chemical Society. E) Microvascular network fusion occurred within multicellular spheres. Reproduced and adapted with permission [183]. Copyright 2009, Elsevier. F) Neovascularization of the middle portion connected the vascular channels on both sides and fluorescent bead perfusion indicated blood flow through the neovascular networks. Reproduced and adapted with permission [190]. Copyright 2021, Springer Nature.

The permeability of vessels is also one of the prerequisites to perform several physiological functions of vessels. The permeabilities of blood vessels for different tissues are completely distinct. For instance, compared to healthy tissues, tumor arteries are more permeable [187]. Currently, vessels constructed by firstly bioprinted and then cell-seeded into the scaffold have an uncontrollable permeability. However, the porous permeability of the hydrogel and cell mixture is controllable. In addition, the permeability of the vessels can also be adjusted by changing the compositions of the bioink [188]. For large diameter vessels, high molecular weight dextran diffusion experiments were used to verify the permeability of the vessels [145]. Compared to the tissues without endothelial channels, the areas with endothelialized vascular channels were found to have a significantly reduced diffusion permeability (Fig. 6A) [151]. It has been found that dextran molecules diffused passively into large vascular channels, then rapidly flowed from capillary junctions into the vascular bed, and finally diffused into the surrounding tissues [171]. More importantly, diffusion permeability was approximately twice as high as it was in the vascular tissue with a capillary network compared to single vessel fluid perfusion (without capillaries). The results on the one hand confirmed the existence of a link between the macro-vascular and micro-vascular systems. On the other hand, it showed the promise of a permeable vascular network system resulting from the integration of artificial vessels to the natural vascular system in the body.

Miller et al. has developed an alveolar model containing a functional vascular topology that was both permeable and perfusable. Interestingly, when oxygen and red blood cells (RBCs) were introduced into the vasculature, the branching topology and hydrogel swelling may promote uniform mixing of blood within the vasculature, allowing rapid oxygen uptake by RBCs [191]. This is an important discovery since lots of current studies have focused on testing the permeability and perfusability of the vasculature but have neglected to verify the oxygen transplantabilty of fresh RBCs. This may be limited by the ratio of oxygen to RBCs and the maintenance of RBCs’ activity in vitro. Thus, permeability and perfusability is the first step to construct the functional vessels in vitro. Since the fundamental purpose of making vessels is to provide sufficient oxygen and nutrients to surrounding tissues, consideration must be also taken to the abilities of ECs on the vessels to selectively absorb oxygen and even small molecules of nutrients after fresh blood is perfused into the vessels.

Perfusion channels play a crucial role in supplying oxygen to large-sized and high cell density tissues. Perfusability implies structural integrity of the vasculature and is also a prerequisite for permeability. In general, most large diameter vessels could be tested in the perfusion experiments (Fig. 6B–C) [157,189]. However, it is difficult to perform direct perfusion due to their extremely small diameters in capillaries. Indeed, many researches have confirmed that the capillary networks can be formed within the bioprinted tissues if ECs in hydrogels undergo angiogenic sprouting and further formed the luminal structures. These capillary networks are often incomplete and disconnected, which may not be processed for perfusion in vitro (Fig. 6D–E) [169,183]. But the researchers induced the formation of a microvascular network that accomplished perfusion by modulating cell adhesion and the degradability of the hydrogel. The vessels could be perfused with 4 μm fluorescent beads, which indicated that it possessed good integrity and perfusability (Fig. 6F) [190]. In particular, the microfluidic model here provided a better design for a vascularized tissue culture system, which also offered the possibility of the applications for bioprinting to create perfusable microvascularized tissue structures with good permeability.

### 5.2 Mechanical strength

For artificial vessels, it is required to match the natural properties of the grafted natural vascular network, such as tensile properties, suture retention, compliance, bursting strength and compression resistance [143]. It is generally difficult to determine the mechanical strength of capillaries because they are single-layered tubular structures formed by spontaneous induction. On the other hand, arteries and veins have larger diameters and the requirements of their mechanical strength are generally higher than those of capillaries [192]. All artificial vessels require a functional EC layer, as it provides a smooth and thrombus-resistant inner surface that facilitates blood flow. However, due to the soft quality of the freshly printed single layer of ECs, the mechanical strength of the printed vascular structure is quiet low. It is often necessary to wait until the tissue structure has sufficient mechanical strength before it is further perfused [171]. Generally speaking, artificial blood vessels need to be stretchy and strong enough to withstand hemodynamic pressures to carry fluids such as the blood flows [193]. Also, if there is a mismatch between the mechanical strength of the vascular graft and the natural tissue, an aneurysm can be triggered [194]. In conclusion, the vascular anastomosis is possible to achieve when the vessel has excellent mechanical properties for transplantable blood vessels.

Bioprinted structures containing cells are typically subjected to static culture within a thermostat, involving regular medium changes. Unfortunately, there are imposed limitations on nutrient and oxygen exchange as well as waste removal [195]. Recognizing that the biomechanical conditions profoundly impact cell growth and proliferation, it becomes imperative to adopt dynamic culture that can provide the requisite mechanical conditions essential for cellular development, including the application of radial and circumferential forces guiding cells in the deposition of ECM [196]. The vascular structure, resembling natural blood vessels in the whole structure and exhibiting enhanced biomechanical properties, underpins the importance of these dynamics [197]. In the quest to emulate physiological conditions, dynamic cultures are routinely executed within bioreactors, where mechanical stimulation devices take charge of regulating internal pressure, flow rates, nutrient supply, oxygen levels, temperature, and pH [4,126]. A comprehensive compilation has been listed in Table 6. Although, most cultures of bioprinted vessels have not been able to achieve dynamic culture with above conditions, many studies have used perfusion culture to achieve endothelialization of large diameter vessels [198]. Moreover, dynamic culture enhances the mechanical properties of bioprinted vascular tissues [25] and fosters the maturation of vascular networks [177]. In conclusion, it is advisable to culture bioprinted vascular structures within bioreactors. This practice significantly promotes cell growth and maturation. Additionally, it ultimately leads to the transformation of these structures into fully functional vascular tissues, largely facilitated by the deposition of tissue-specific ECM.

The main functions of the vasculature are dependent on the regulation of tissue composition and the growth (including remolding) of the tissue structures, which ensure their mechanical homeostasis [143]. In natural blood vessels, SMCs contribute significantly to the elasticity, ductility, and mechanical properties of the vessels [186]. The growth and remodeling process of vascular tissues ensures their mechanical homeostasis. At the tissue level, vessels maintain their structures and mechanical strengths through the massive deposition of ECM and the degradation of biomaterials [199]. Recent studies has demonstrated that bioprinted vessels containing only fibroblasts showed increased modulus of elasticity, rupture pressure, and ultimate tensile strength after prolonged incubation. Although it was not functionally sufficient to mimic a vein, the large amount of collagen made the physical properties of the vessel almost similar to those of a vein [200]. It is speculated that artificial vessels needs to accumulate sufficient ECM-associated proteins in order to be mechanically strong to resist the high frictional forces associated with fluids.

Protein-based or polysaccharide-based hydrogels are often used to simulate the action of ECM [44]. Despite their good biocompatibility, due to the low stiffness of these hydrogels, large diameter bioprinted vessels created by them can collapse and fail to take shapes due to gravity. Three means are often used to increase the mechanical strengths of these vascular tissues:

(1) Adding nanomaterials or polymers to improve the mechanical properties of the vessel [182]. Silicone resin can be used as a biological scaffold because of its excellent mechanical strength and good permeability. Xu et al. [181] mixed dECM and silicone to bioprinted tubular structures with elastic modulus close to that of the natural aorta.
(2) Combining extrusion-based bioprinting with rotational longitudinal extrusion to produce a complete large-diameter vascular structure [199,[184], [201], [202], [203]]. This approach can avoid the deformation of the bioink during layer build-up.
(3) Using a scaffold with high stiffness, such as polycaprolactone [157,203,204]. The stiff scaffold acts as a support for the vessels in the soft hydrogels, allowing the vessel structure to be fixed into shape and avoiding deformation of the vessel during the culture process.

So far, there is almost no perfect bioprinting method that can produce functional vessels with good mechanical strength, as it is extremely challenging to maintain a balance between the mechanical strength of the vessel structure and the cellular activity. Most hydrogels used have a moderate degree of crosslink density. These hydrogels can maintain the shape of the tube during fabrication and ensure that the fidelity of the vessel is maintained and that the overall mechanical properties support the growth of vascular-associated cells [192]. Under ideal conditions where the shape of the vessel does not change and the cells remain highly active, the cells embedded in the vessel secrete large amounts of ECM proteins as the in vitro culture time increases and the surrounding hydrogel is gradually degraded. Eventually the engineering vessel forms a mechanically strong vascular structure with a similar mechanical property to the natural/grafted vascular systems.

### 5.3 Vascular anastomosis

**Fig. 7:**
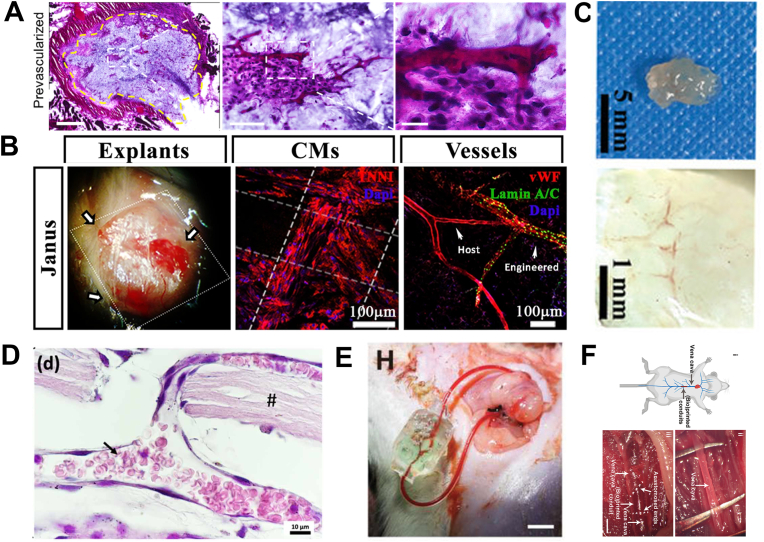
**Transplantation of the bioprinted vascular tissue where vascular anastomosis and perfusion occurred.** A) The pre-vascularized tissue has a large number of endothelial vessels with RBCs, indicating successful anastomosis of the artificial vessel with the host vessel. Reproduced and adapted with permission [29]. Copyright 2017, Elsevier. B) Integration of the human-derived vascular network with the host vascular network. Reproduced and adapted with permission [206]. Copyright 2018, Springer Nature. C) Bioprinted loaded scaffolds (HUVECs-3LMS-GelMA) induce vascular growth after 4 weeks of subcutaneous implantation, exhibiting durable angiogenic properties. Reproduced and adapted with permission [38]. Copyright 2023, John Wiley and Sons. D) The assembled vascular system was strongly bound to the host tissue and abundant blood perfusion was observed both in the peripheral and central regions. Reproduced and adapted with permission [162]. Copyright 2017, Institute of Physics Publishing. E) Successful perfusion of blood flow following the establishment of anastomoses between the grafted tissue and the carotid artery as well as jugular vein. Reproduced and adapted with permissio [146]. Copyright 2021, John Wiley and Sons. F) Bioprinted conduits enable in vivo implantation into mouse vena cava and achieve perfusion through vascular anastomosis. Reproduced and adapted with permission [11]. Copyright 2022, American Association for the Advancement of Science.

Alexis Carrel firstly introduced vascular anastomosis [205], which paved the way for organ transplantation. Traditionally, vascular anastomosis refers to the direct connection between artery and artery, vein and vein and even artery and vein. So that the vessels of the foreign graft are tightly connected to the natural blood vessels in the body and the fresh blood can be supplied to the graft. For bioprinted constructs, the key for successful transplantation is that vascular structures can rapidly conform to the transplanted body (i.e. the artificial vessels are well remodeled to ensure that these artificial ones can quickly interoperate with the native vessels in the body). Anastomoses between different vessels can be achieved by multiple strategies. Venous anastomoses is achieved using vascular anastomoses; while arterial anastomoses is achieved mainly by surgical sutures [202]. Due to the lack of mechanical strength, most current bioprinted vessels cannot meet the criteria for either venous or arterial anastomosis, but some vascularized tissue structures have been transplanted to demonstrate the feasibility of the anastomoses for capillary network (Fig. 7A–C) [29,38,206]. Oliveira et al. [162] transplanted a mixture of fibroblasts and HUVECs cultured for four weeks into the subcutis of immune-deficient mice (Fig. 7D). Three weeks after transplantation, the vessels successfully connected to the host circulatory system and the foreign cells had undergone rapid fusion with the host vessels**.**

The anastomosis of vascularized tissues was achieved in the study by Xin Liu et al. who fabricated vascularized liver tissue to grow neovascularization in the peritoneum of mice (Fig. 7E) [146]. Although they attached the tissue to the arteries of experimental animals in an artery-vein pattern and blood could be perfused, problems such as occlusion of blood vessels due to thrombosis needed to be avoided by adding heparin periodically during the procedure. For large/small caliber vessel transplantation, the bioprinted conduit was grafted into the vena cava and blood perfusion was achieved, demonstrating the potential for the in vivo applications of revascularization (Fig. 7F) [11]. Though this developing vascular network will continue to grow within the body, fast fusion with the native vasculature is impossible here. It has been proven that it took a few days/weeks for the integration of the vascularized structures with the host vasculature [189]. In conclusion, the integration and the anastomosis of vascular tissues to the natural vessel systems always require that the vessels have been cultured to a certain level of maturity in vitro before they can be transplanted in vivo.

Vascular maturation is influenced by the environment after transplantation and is not solely determined by culture time. In one study, subcutaneous transplantation of vascularized xenomas with hydrogel microfibers that had been cultured in vitro for only one week was found to generate neovascularization with hybridized human and mice endothelial-like cells [163]. It has been demonstrated that the initial shape of the neovascularization did not affect the vascular remodeling process [165]. The tissue deformation may also be an external environmental factor influencing vascular remodeling. ECM environment in turn influences the process of tissue deformation. Therefore, the natural tissue culture environment also further promotes neovascularization and vascular integration when mature vascularized tissue is transplanted in vivo.

## 6 Evaluations and applications of vascular tissues at the organ level

Bioprinted vascular structures exhibit the ability to support the growth of non-vascularized tissues or to use in vascular transplantation. Vascular grafts used in transplantation need to be tubular in nature, exhibiting a bilayer structure composed of smooth muscle cells on the outer surface and ECs encircling the lumen for perfusion. In addition to their perfusability, they should also possess biological functions such as quiescence and contractility in response to vasoconstrictor agents [207]. For transplantable vascular grafts, assessment of biologic properties of artificial vessels is crucial to prevent issues such as degradability, collapse, or restenosis. These properties include sufficient mechanical strength, physical stability, and cell preservation stability. However, it should be noted that bioprinted vascular constructs have not been successfully transplanted into human body. Nonetheless, vascularized tissue structures at the organ level need to have the similar physiological functions both in vitro and in vivo.

**Fig. 8:**
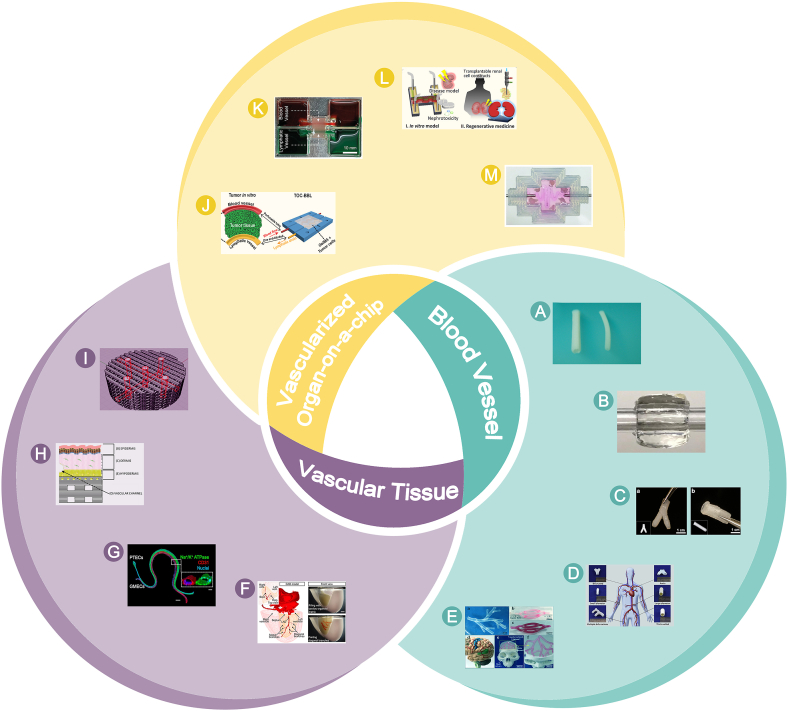
**Applications of bioprinted vascular structure.** A)-B) Simple large diameter vascular structures. Reproduced and adapted with permission [189]. Copyright 2009, Elsevier. Reproduced and adapted with permission [119]. Copyright 2019, Elsevier. C) Branched and linear vessels. Reproduced and adapted with permission [208]. Copyright 2022, American Chemical Society. D)-E) Complex multi-branching vascular structures. Reproduced and adapted with permission [204]. Copyright 2019, Elsevier. Reproduced and adapted with permission [208]. Copyright 2017, American Chemical Society. F) Biofabricated heart structures. Reproduced and adapted with permission [209]. Copyright 2019, American Association for the Advancement of Science. G) The renal model simulates albumin uptake and glucose reabsorption in vitro. Reproduced and adapted with permission [210]. Copyright 2019, Proceedings of the National Academy of Sciences. H) Skin model for dermatological modeling and wound healing. Reproduced and adapted with permission [211]. Copyright 2018, John Wiley and Sons. I) Bone tissue enhances osteogenic bone regeneration and vascular cell growth. Reproduced and adapted with permission [212]. Copyright 2016, Institute of Physics Publishing. J) Biomimetic tumor model to study drug diffusion behavior. Reproduced and adapted with permission [188,213]. Copyright 2019, John Wiley and Sons. K) Bionic Vascular and Lymphatic Vessel Models Containing Melanoma Spheroids. Reproduced and adapted with permission [214]. Copyright 2022, Wiley-VCH GmbH. L) Proximal renal tubule chip composed of mature renal tubules and vascular parts. Reproduced and adapted with permission [215]. Copyright 2020, Elsevier. M) Tumors model within a vascularized construct. Reproduced and adapted with permission [179]. Copyright 2018, John Wiley and Sons.

Bioprinting technology, with its high precision and controllability, allows for the fabrication of various vascular structures with different diameters using bioink-supported cells to exhibit their three-dimensional morphology. These vascular structures mainly consist of mm-scale vessels containing dual layers of vascular cells, mm-scale vessels with multilayer structures and μm-scale microvasculature with complex capillary networks. Since distinct vascular functions are required in different biological applications, it is necessary to fabricate the above complex vascular system composed of three types of vessels with diverse functions. Fig. 8 and Table 8 show the application of functional organ models of vascular/vascularized tissues and vascular chips at organ level.

**Table 8:** Application of functional organ models.

Organ models	Vascular tissue	Performance of function	Application prospects	Ref.
Vessel models	Three-layer vascular structure	Secreting type IV collagen/Forming monolayer ECs and typical network structure	Building in vitro models of tumour and angiogenesis	[152]
	Double-layer small diameter vessels	Appearing a clear border between the SMC and fibroblast layers	Constructing a three-layer vascular structure	[189]
	Bifurcated vascular structures	High cell survival rate	Cellular heterogeneous multilayered tubular structures	[216]
	Single-layer tubular construction	Adequate mechanical strength	Functional vascular structure	[184]
	Double-layer tubular construction	Recreating the geometry of human blood vessels/thrombotic blockages	Studying vascular thrombosis and inflammatory responses	[217]
	Single layer large diameter tubular construction	The burst pressure: approximately 52 % of the human saphenous vein	Reproducing veins with mechanical strength	[199]
Vascular tissue models	Heart Patch	Retaining contractility when transplanted into the body	Manufacture of clinically relevant cardiac patches	[218,219]
	Liver tissue	Successful vascular anastomosis: hydrogel liver tissue permeated by host blood	No data	[191]
	Skin tissue	Formation of mature perfusable vascularized channels	Skin diseases model	[[211], [220], [221]]
	Vascularized proximal tubule model	Albumin uptake/glucose reabsorption	Studying kidney function/disease modeling and pharmacology	[210,222]
	Bone tissue	Greatly improving nutrient transport/maintaining cellular viability/promoting differentiation of osteoblasts	Study of the process of bone tissue differentiation and bone repair	[151,[212], [223], [224], [225]]
	Intestinal tissue	Co-culture of intestinal epithelial cells with ECs	Study of relevant biological phenomena	[213]
	Vascularized multicellular spheroids (Embryoid EB/neurospheres/cardiac spheroids)	Perfusion-ready heart tissue synchronised for fusion and beating within seven days	Rapid assembly of perfusable organ-specific tissue	[209]
Vessel-on-a-chip(Vascular organ chips)	Vascularized proximal kidney chip	Implanted into the host kidney tissue through infiltration of the host blood vessels	Accurate predictive tools for drug development, drug screening or disease modeling	[215]
	Blood clot model	Reproducing the process of thrombosis	Studying thrombosis, thrombolysis and fibrosis	[226]
	Bionic tumor model	Simulating drug transport in the tumor microenvironment	Cancer drug screening	[188]

### 6.1 Vascular structure with potential as grafts

Over the past decade, there are still giant challenges for the successful transplantation of bioprinted vascular tissue into body. There are several reasons: (1) The transplanted vessels do not match the natural ones; (2) Inadequate ECM secretion leads to insufficient mechanical strength; (3) It is difficult for different bioinks (containing biomaterials and cells) to be successfully bioprinted and to fuse together effectively after bioprinting; (4) There are limited designs to make ECs and SMCs have enough interactions with each other to form mature large vasculatures. All the above deficiencies illustrate the high demands on the development of culture environments and bioinks, which require the robust supports from the innovating bioprinting technologies and the culture systems.

In general, the blood vessels needed for transplantation in surgery were larger than 1 mm in diameter [144]. In contrast, bioprinting technology has achieved micron-level precision. Thus, bioprinting technology can be used to fabricate the customized vascular structures for patients in theory. While bioprinted vascular tissue may not fully replicate the functions of natural blood vessels, they serve as valuable tools in medical studies. Vascular structures that can constrict have been constructed to mimic vascular tissue in diseased states such as intimal hyperplasia, atherosclerosis and cardiovascular stenosis, thereby advancing our understanding of cardiovascular diseases (CVDs) [103]. In another study, bioprinted vessels resembling natural arteries were utilized to investigate the vascular response to viral infection and to evaluate the effectiveness of antiviral drugs through conduit constriction and dilation [11].

In the field of bioprinting, the absence of standardized criteria for transplantable bioprinted vascular tissue is lacking. Prior to transplantation, it is essential to thoroughly evaluate the performance of vascular grafts through in vitro testing, including comprehensive mechanical characterization such as tensile testing to determine parameters like modulus of elasticity or storage modulus. Referring to the criteria proposed by the FDA regarding transplantable artificial vessels [194], some of the following indicators may be used for assessing bioprinted vascular tissue functions. In addition to the biocompatibilities and aseptic grafting, thrombosis, embolic events, vessel occlusion with stenosis, allergic reactions and aneurysm formation all need to be avoided. Jang et al. [203] implanted triple-layered artificial vessels into bilateral carotid and femoral arteries in dogs and found vessels with little or no fibrin clots and even no acute thrombosis formed after graft bypass surgery, demonstrating the considerable functions of the vessels at organ level.

### 6.2 Microvasculature for biological research model

Vascular networking plays a crucial role in enabling organs to perform their normal physiological functions. The research on vascularized tissue engineering is rapidly advancing, primarily focusing on the in vitro construction of vascularized organ/tumor models. Notable successes have been achieved in the development of organ models including skeletal muscle models [208], islet models [40], hepatic sinusoidal-like models [94], and meniscus structures [41]. However, fabricating osteomimetic scaffolds for bone tissue models remains challenging due to the complexity and high stiffness of microchannels involved in angiogenesis, osteogenesis, and mechanical tests. A recent study addressed these challenges by constructing multi-microchannel and vascularized bone tissues that replicated the central medullary canal, peripheral Haversian canal, and transverse Volkmann canal of bone structures, resulting in accelerating bone defect repair [227]. Kuo et al. [228] constructed an embryonic model that simulated trophoblast-EC interactions in vitro, which held the promise of further understanding the pregnancy-related pathology and expanding treatment strategies. These advanced organ models offered valuable insights into cellular interactions and can simulate therapeutic effects for improved safety and efficacy in vivo transplantation.

Incorporating bioprinting technology, tumor models have gained significant attention due to their broad range of applications. Firstly, they can recapitulate key events in tumorigenesis, such as tumor-mesenchymal interactions, tumor invasion, and in vivo infiltration. Additionally, these models facilitate the exploration of pathological mechanisms underlying tumors and the evaluation of targeted anti-cancer therapies in vitro. Moreover, tissue models combining patient cancer cells and the tumor microenvironment enable the assessment of patient-specific drug responses, leading to more accurate monitoring of cancer drug efficacy. Notably, recent advancements in bioprinted tumor models have shown promising progress. For instance, Won-Woo Cho et al. [147] developed a blood-lymphatic integration system comprising heterogeneous melanoma spheroids (BLISH): bionic vessels (bv) and lymphatic vessels (lv). This model has contributed to enhancing our understanding of melanoma progression and improving the potential transplantabilty of cancer treatments. Furthermore, lung cancer models have been established to facilitate the development of targeted therapies and anti-tumor drugs [61].

### 6.3 Vessel-on-a-chip

Organ-on-a-chip refers to a microfluidic device containing organ-specific cells used to model their structures and functions at the organ level [229]. The vascular network containing ECs involved in the organ-on-a-chip system is the basis for ensuring vascular-organ interactions. The integration of the vascular system into the chip can reconstruct the microenvironment and physiological functions of the organ.

Currently, organ-on-chips are mainly produced through photolithography and soft lithography, but they may also be produced using bioprinting [230]. The utilization of vessel-on-a-chip technology enables the investigation of physiological processes in tissues and organs, as well as the mechanisms of drug action, in vitro within a conducive, three-dimensional ECM microenvironment. Some researchers have combined extrusion bioprinting to develop an advanced proximal renal tubule chip with vessels containing ECs, which are necessary to simulate physiological functions such as renal reabsorption in vitro [215]. Zeming Gu et al. constructed perfusable vessel-on-a-chip as a model for anti-angiogenic drug screening [123].

In addition, vessel-on-a-chip has the capability to replicate pathological processes associated with disease onset, particularly tumorigenesis. Nothdurfter et al. [42] presented the first model of a microvascularized tumor environment that was directly bioprinted onto a fluidic chip. Yu et al. have constructed a bionic tumor model including a microcirculatory (vascular and lymphatic) system to study drug diffusion and to deliver different drugs by perfusing to evaluate tissue sensitization [182]. Besides, bioprinted chips can mimic pathological processes such as thrombosis. Khademhossein's group has developed a novel in vitro thrombosis chip platform that can be used as a model for studying thrombosis, thrombolysis and fibrosis [226].

In recent years, several large-diameter blood vessels have been fabricated; however, there remains a scarcity of structures with substantial vascular binding tissues. To address this issue, it has been proposed to employ multiple small fabrication modules to construct larger vascular structure tissues. Hyoryung Nam et al. [42] successfully developed an in vitro model for respiratory diseases and achieved the reconstruction of a perfusable vascularized trachea model (TM) at the interface between blood vessels and tracheal epithelium. This model was designed to simulate inflammatory responses and can even be utilized for drug testing to mimic systemic inflammation. Similarly, the use of multiple perfusion chips can offer more significant advantages compared to a single organ chip. By connecting them in tandem, it becomes possible to simulate interactions between different tissue structures and thereby to study the effects of upstream products on downstream components, leading to the results that are more biologically relevant.

While these paradigms may not fully replicate the function of most vascular tissues and networks in vivo at the cellular, tissue, and organ levels, they hold tremendous potential for investigating the mechanisms underlying human diseases. Vascularized tissues can be used as the models for the following diseases such as inflammation, immune response and tumor angiogenesis [145]. This will enable more effective screening of new therapies and the design of the personalized drug therapies. The use of bioprinted vascular tissues will enable to construct various types of vascular-related organ-on-a-chip models, including the endothelial barrier models, which will be widely used to model pathological processes and drug screening or disease diagnoses [229].

## 7 Summary and outlook

Although much progress has been made in the field of bioprinting for in vitro manufacturing of artificial blood vessels, bioprinted blood vessels cannot be used for clinical treatments such as in vivo transplantation. There are several challenges that need to be addressed:

(1) For single-layer vascular structures: although bioprinting can replicate large-diameter vascular structures, they are always initially soft [196]; and it is critical to deposit sufficient ECM proteins (e.g., collagen) to support the stiffness of the vessels.
(2) For multilayered vessels: achieve circumferential alignment of SMCs in the smooth muscle layer and dynamic culture to recapitulate the required structures and functions.
(3) For vascular network structures: the resolution of bioprinting needs to be improved and optimal external culture conditions are required to induce spontaneous formation of capillary networks.

To overcome these difficulties, selecting a suitable bioink that can better simulate the growth microenvironment of blood vessels is firstly needed. The formulation of bioink should be designed based on the rheological properties and compatibility with biological materials. The choice of bioprinting technique should align with the characteristics of the printer and the cross-linking principle of the hydrogels. Moreover, due to the lack of indicators for assessing the functions of in vitro cultured vascular structures, this review emphasizes the importance of function indicators at different stages: cellular, tissue, and organ levels.

While there are still limitations in fabricating functional blood vessels, such as limited cell type diversity available in studies, challenges for inducing angiogenesis in multi-scale vascular networks through growth factor gradients, and difficulties for co-development and functionalization of large vessels with small vessels and microvessels. However, numerous studies have demonstrated its wide application in biological research. Biomanufacturing of mechanical and functional relevant vessels has the potential to serve as a vascular model for studying diseases in vitro and as surgical grafts in vivo, with broad biomedical applications in the future.

Besides, vascular biomanufacturing requires interdisciplinary support and collaboration among researchers, clinicians, and regulatory agencies. This collaboration is essential for constructing fully functional and transplantable vessels. Autologous revascularization remains the gold standard for cardiovascular disease treatment, and it is crucial that artificial vessels enable successful vascular anastomosis with the transplanted individual. However, vascular anastomosis is only the first step towards successful transplantation. The physiological and mechanical factors need to be considered while constructing vascular structures suitable for in vivo implantation.

With advancements in bioink and bioprinting technologies, bioprinted manufactured blood vessels are expected to contribute significantly to the medical field. To meet practical needs, the differences and consistencies between bioprinted vessels and in vivo blood vessels should be evaluated at three levels: (1) biomimetic vascular layered structures for appropriate functionalization; (2) micron-scale functional capillaries as clinical therapeutics; (3) precise generation of complex layered vascular networks that match host tissues. Additionally, the combination of bioprinting and organ-on-a-chip technology enables the construction of 3D tissue models with intricate microvasculature. It provides the possibility of manufacturing patient-specific blood vessels or vascular chips to build patient-specific in vitro physiological or disease models, which can facilitate a deeper understanding of disease research mechanisms, including cancer and cardiovascular disease studies.

## Declaration of competing interest

We declare that we have no financial and personal relationships with other people or organizations that can inappropriately influence our work, there is no professional or other personal interest of any nature or kind in any product, service and/or company that could be construed as influencing the position presented in, or the review of, the manuscript entitled, “Bioprinted vascular tissue: assessing functions from cellular, tissue to organ levels”.

## Data Availability

No data was used for the research described in the article.
